# Co-Localization of the Oncogenic Transcription Factor MYCN and the DNA Methyl Binding Protein MeCP2 at Genomic Sites in Neuroblastoma

**DOI:** 10.1371/journal.pone.0021436

**Published:** 2011-06-22

**Authors:** Derek M. Murphy, Patrick G. Buckley, Sudipto Das, Karen M. Watters, Kenneth Bryan, Raymond L. Stallings

**Affiliations:** 1 Department of Cancer Genetics, Royal College of Surgeons in Ireland, Dublin, Ireland; 2 Children's Research Centre, Our Lady's Children's Hospital, Crumlin, Dublin, Ireland; 3 Department of Molecular and Cellular Therapeutics, Royal College of Surgeons in Ireland, Dublin, Ireland; City of Hope National Medical Center and Beckman Research Institute, United States of America

## Abstract

**Background:**

MYCN is a transcription factor that is expressed during the development of the neural crest and its dysregulation plays a major role in the pathogenesis of pediatric cancers such as neuroblastoma, medulloblastoma and rhabdomyosarcoma. MeCP2 is a CpG methyl binding protein which has been associated with a number of cancers and developmental disorders, particularly Rett syndrome.

**Methods and Findings:**

Using an integrative global genomics approach involving chromatin immunoprecipitation applied to microarrays, we have determined that MYCN and MeCP2 co-localize to gene promoter regions, as well as inter/intragenic sites, within the neuroblastoma genome (MYCN amplified Kelly cells) at high frequency (70.2% of MYCN sites were also positive for MeCP2). Intriguingly, the frequency of co-localization was significantly less at promoter regions exhibiting substantial hypermethylation (8.7%), as determined by methylated DNA immunoprecipitation (MeDIP) applied to the same microarrays. Co-immunoprecipitation of MYCN using an anti-MeCP2 antibody indicated that a MYCN/MeCP2 interaction occurs at protein level. mRNA expression profiling revealed that the median expression of genes with promoters bound by MYCN was significantly higher than for genes bound by MeCP2, and that genes bound by both proteins had intermediate expression. Pathway analysis was carried out for genes bound by MYCN, MeCP2 or MYCN/MeCP2, revealing higher order functions.

**Conclusions:**

Our results indicate that MYCN and MeCP2 protein interact and co-localize to similar genomic sites at very high frequency, and that the patterns of binding of these proteins can be associated with significant differences in transcriptional activity. Although it is not yet known if this interaction contributes to neuroblastoma disease pathogenesis, it is intriguing that the interaction occurs at the promoter regions of several genes important for the development of neuroblastoma, including *ALK*, *AURKA* and *BDNF*.

## Introduction

*MYCN* is a member of the *MYC* family of basic helix-loop-helix (bHLH) transcription factors which regulate a diverse range of cellular processes including proliferation, differentiation and apoptosis [1]. High level amplification of *MYCN* occurs in multiple pediatric cancers, and for neuroblastoma it is the most important genetic prognostic indicator of poor clinical outcome [2]. Further evidence that this transcription factor directly contributes to tumorigenesis is provided by the development of neuroblastoma-like tumors in a transgenic mouse model over-expressing MYCN [3].

MYC family members heterodimerize with MAX at DNA target sequences known as E-boxes, recruiting histone acetyltransferases (HAT) and activating gene expression [4]. MYC proteins have also been shown to act as transcriptional repressors by association with MIZ1 and function through the inhibition of SP1 activity [5], [6]. Previously, we demonstrated that MYCN has a significantly greater affinity for the *CATGTG* motif than for *CACGTG*, which is significantly associated with c-MYC binding sites [7], [8]. We further demonstrated that aberrantly high levels of MYCN promote the occupancy of weaker affinity E-box elements, thus commandeering the functional role of other transcription factors [9]. Aberrant target sites for MYCN binding are highly enriched for genes that regulate aspects of the cell cycle, leading predominantly to the up-regulation of these genes [10]. In addition to directly affecting the expression of genes [9]–[16] and miRNAs [17]–[20] by direct binding to promoter regions, changes in MYCN levels undoubtedly also cause a cascade of secondary alterations to the transcriptome.

Genome-wide analysis of MYCN binding has also established a more global role for MYCN as a mediator of chromatin structure [21]. Consistent with this concept, we recently reported the association of MYCN to regions of DNA hypermethylation, through the integration of chromatin immunoprecipitation on chip (ChIP-chip) and methylated DNA immunoprecipitation on chip (MeDIP-chip) data [9]. The aberrant hypermethylation of gene promoters is a well known mechanism for the transcriptional silencing of tumor suppressor genes in many forms of cancer including neuroblastoma [22]–[24]. We hypothesized that the association between MYCN binding and regions of DNA hypermethylation may be due to the action of an intermediate methyl binding protein (MBD). Of the MBD proteins, MeCP2 is essential in human brain development and has been linked to several cancer types and neurodevelopmental disorders [25]–[28]. MeCP2 can selectively bind to methylated CpG residues, has been shown to localize to inactive heterochromatic regions of DNA, and interacts with the transcriptional repressor SIN3A to recruit histone deacetylases (HDAC) to repress transcription of methylated promoters [29], [30].

However, this classic model of MeCP2 as a transcriptional repressor has been called into question, as methylated and imprinted genes remain silent in MeCP2-deficient mice [31] and gene expression profiling experiments failed to identify MeCP2 target genes regulated by methylated promoters [32]. A genome-wide study of MeCP2 binding sites revealed that a large percentage of MeCP2-bound gene promoters were unmethylated and actively transcribed [33]. This result was supported by Charhour *et al.* [34] in a study showing the interaction of MeCP2 with the transcriptional activator CREB1 at active promoters. Additionally, a new model of MeCP2 function proposes that MeCP2 can act as a transcriptional modulator, regulating chromatin structure at distal methylation sites to modulate the expression of active genes [35]. (For reviews, see [28], [36])

In this study, we have carried out genome-wide analysis of MYCN and MeCP2 binding sites in combination with methylation analysis, and have characterized a novel co-occupancy of these proteins at promoter regions. Gene expression analysis of bound promoters reveals differential expression levels of genes bound individually by, or in combination with MYCN and MeCP2.

Taken together, our results suggest that the majority of hypermethylated MYCN sites are also bound by the methyl binding protein MeCP2, that a greater number of MYCN/MeCP2 positive sites occur outside of hypermethylated loci and points to a role for MeCP2 in the modulation of gene expression in MYCN amplified tumors.

## Results

### Co-occupancy of Genomic Regions by MYCN and MeCP2

We previously reported that MYCN co-localizes to regions of hypermethylated DNA in neuroblastoma cell lines at a significantly higher than expected frequency [9]. Here, we test the hypothesis that this association might be due to the interaction of MYCN with MeCP2, which is capable of directly binding to methylated DNA and is known to play a role in cancer and neurodegenerative disorders [26]–[28]. For an initial assessment of this hypothesis, chromatin from the ***MYCN*** amplified neuroblastoma cell line Kelly was immunoprecipitated with an anti-MeCP2 antibody and then hybridized to the NimbleGen HG18 two-array promoter set and to a custom designed tiling array representing 528 miRNA loci, as described previously [9]. In order to determine the extent of MYCN and MeCP2 co-occupancy to regions of hypermethylation, MeDIP-chip was also performed on the Kelly cell line, using the above array platforms.

The MeCP2 ChIP-chip experiments were carried out in duplicate on both the HG18 two-array promoter set and the custom tiling array, and as illustrated in Figure S1A, B and C, there was a high correspondence between the biological replicate experiments for each microarray (r = 0.89 and 0.92 for the two array promoter set; r = 0.88 for the custom tiling array). For further validation of the MeCP2 ChIP-chip experiments, qPCR primers were designed for seven randomly selected regions showing enhanced MeCP2 binding on the ChIP-chip experiments (Table S1). Six out of seven qPCR experiments showed >1.5 fold enrichment of DNA sequence from the MeCP2 immunoprecipated sample relative to the IgG negative control, indicating that the microarray results were of high quality (Figure S1D). Comparison of our data to previously published MeCP2 target sites [34], [37] confirmed the presence of positive MeCP2 sites at the promoter regions of *SST, MEF2C, GPRIN1* and *SGK* (Figure S2 A–D). In an additional study, Yasui *et al.* [33] performed ChIP-chip analysis of MeCP2 precipitated DNA from the neuroblastoma cell line SH-SY5Y using a custom designed microarray which tiled 26.3 Mb of imprinted and non-imprinted regions. In total, twelve positive promoters from this data set were selected and examined for MeCP2 binding in our results. Of these, eight were positive for high confidence MeCP2 binding sites (Figure S2 E, F and Table S1). The discordance found between data sets could be due to genuine biological differences between the cell lines used or technical issues such as the use of different MeCP2 antibodies for immunoprecipitation. The results from MYCN ChIP-chip and MeDIP experiments have also been rigorously validated, as detailed previously [9].

**Figure 1:**
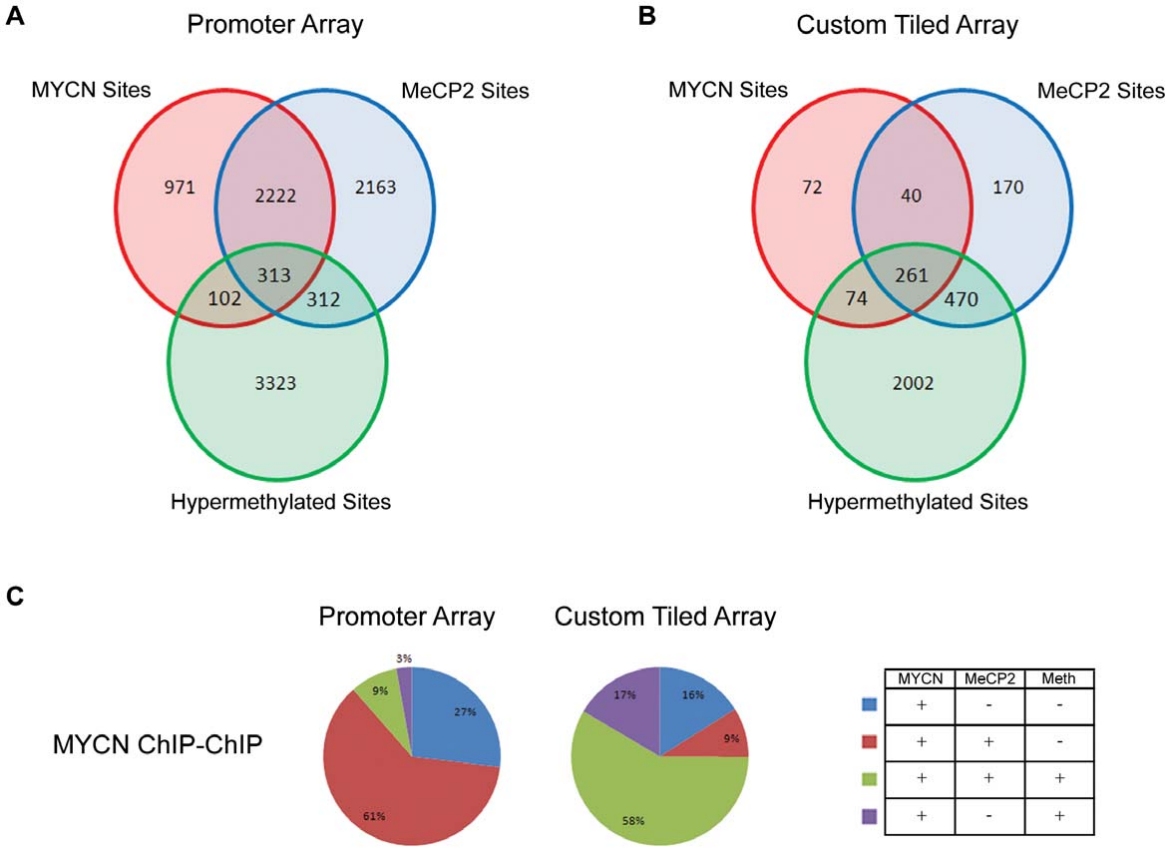
Genome-wide analysis of MYCN and MeCP2 binding sites. (A) & (B) Three-way Venn diagrams displaying the number unique and overlapping binding sites from MYCN and MeCP2 ChIP-Chip experiments along with the hypermethylated sites identified from MeDIP analysis of the Kelly cell line, hybridized to (A) the two-array promoter set and (B) custom tiled arrays (B). (C) Pie charts representing the percentage of MYCN sites which are unique to the MYCN dataset and which overlap sites enriched for MeCP2 binding and regions of hypermethylation.

Data generated from MYCN, MeCP2 ChIP-chip experiments and MeDIP-chip experiments were analyzed using a custom *Java* application to determine the extent of overlap between data sets. Figure 1A depicts the number of significant MYCN, MeCP2 and methylation sites which were in common and unique to each data set from the promoter arrays, which includes an average coverage of 4.7 kb around promoters for all annotated genes from RefSeq, UCSC and the Mammalian Gene Collection. Similar to our previous finding with the CpG Island/promoter array data set [9], ∼11.5% (n = 415) of MYCN sites were associated with hypermethylated promoters, representing a statistically significant enrichment of MYCN at these sites (P<0.001). Approximately 75% (n = 313) of these MYCN bound hypermethylated sites that overlapped with MeCP2 binding sites. Remarkably, the co-localization of MYCN/MeCP2 binding also occurred at promoter regions lacking detectable hypermethylation, with 62% (n = 2,222; P<0.001) of MYCN sites being co-occupied by MeCP2. The percentage of hypermethylated MeCP2 bound sites was evenly split between those uniquely bound by MeCP2 (312 sites) and those co-occupied by MYCN (313 sites) and the vast majority of hypermethylated sites (82%, 3,323 sites) within Kelly are not bound by MYCN or MeCP2.

Figure 1B depicts a three-way Venn diagram of the analyzed custom tiling array datasets for miRNA regions. In contrast to the promoter array results, a significant increase in the percentage of MYCN sites associated with MeCP2 at hypermethylated regions was observed. Of the 447 MYCN positive sites, 58% (n = 261) were co-occupied with MeCP2 at hypermethylated loci, compared with just 9% (n = 313) of MYCN sites identified with the promoter array (Figure 1C). A similar shift was observed for the number of MeCP2 sites associated with regions of hypermethylation. Of the 935 MeCP2 sites, 50% (n = 470) were associated with hypermethylated loci, compared to just 6% (n = 312) of MeCP2 sites identified using the promoter array (Figure S3). The increased association of MYCN and MeCP2 to regions of hypermethylation could be explained in part by the structural functions of these molecules, as the miRNA array contains a significant amount of inter/intragenic sequence.

**Figure 2:**
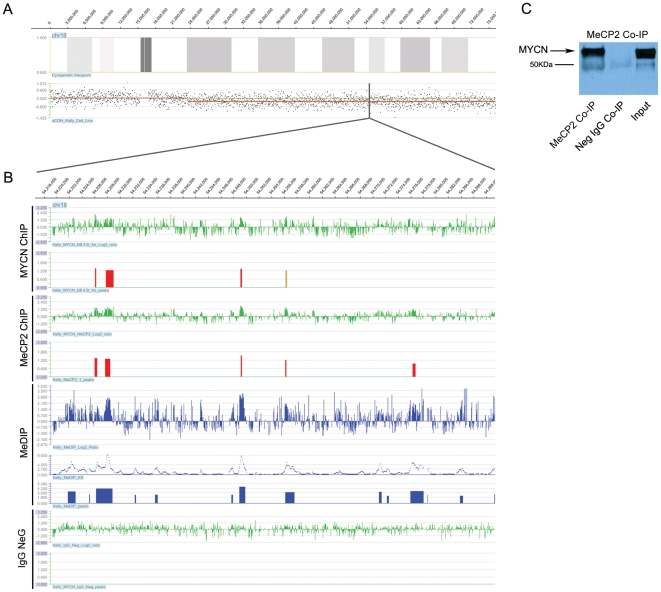
Analysis of MYCN, MeCP2 and hypermethylation in a region of hemizygous deletion. (A) Array CGH analysis of the Kelly cell line showing a large 52.6MB terminal deletion on the long-arm of chromosome 18. (B) A tiled 70.5-kb section of the hemizygously deleted region on chromosome 18, the upper panels display the raw log_2_ ratios and the identified, consistent binding sites for both MYCN and MeCP2. Peaks displayed are considered high confidence binding sites with an FDR score of <0.05 (red peaks) and 0.05-0.1 (orange peaks). Kelly MeDIP results are represented by the panels in blue, including the raw log_2_ ratios, Kolomogorov-Smirnov test p-values (−log_10_), and the identified peaks of hypermethylation. The lower panels show the results of a negative control experiment using normal mouse IgG. (C) A western blot of a Co-IP performed using anti-MeCP2 antibody.

In order to demonstrate that MYCN, MeCP2 and hypermethylation co-localization occurs on the same chromosomal homolog, we examined a terminally deleted region on the long arm of chromosome 18 in the Kelly cell line (Figure 2A). Integration of aCGH, ChIP-chip and MeDIP-chip data revealed an overlap of MYCN, MeCP2 positive and DNA hypermethylated sites confirming that this co-occupancy occurs on the same chromosome homolog (Figure 2B). To confirm specificity of the array data derived from the immunoprecipitation experiments, we performed negative control ChIP-chip experiments using normal mouse IgG. The lower tracks in Figure 2B depict the results of that experiment showing no overlap with the MYCN, MeCP2 and MeDIP sites.

Given that, 75% of hypermethylated MYCN promoter sites were associated with MeCP2, and that 61% of the total MYCN promoter sites were co-occupied by MeCP2 were non-hypermethylated regions, we carried out a co-immunoprecipitation analysis using the MeCP2 antibody on nuclear extract obtained from Kelly cells to determine if the association detected by ChIP-chip data was due to the direct interaction of MYCN with MeCP2 protein. As illustrated in Figure 2C, MYCN co-immunoprecipitated with MeCP2, leading us to conclude that the majority of hypermethylated MYCN sites are also bound by the methyl binding protein MeCP2. The reciprocal experiment, involving MYCN antibody on nuclear extract, was uninformative as it was not possible to resolve MeCP2 by electrophoresis from a similarly sized IgG protein.

### CpG Island Occupancy and Effects on Gene Expression

In the context of gene expression, hypermethylation generally has a negative regulatory impact when occurring at CpG islands within the promoter region of a gene, so it was of interest to compare how the occupancy of MYCN and MeCP2 at such sites impacts upon gene expression [Tables 1]. Among the 4,050 hypermethylated regions identified in Kelly using the two-array promoter set, 1,077 were associated with a UCSC annotated CpG island, representing a significant enrichment of hypermethylation at CpG island sites. Consistent with a previous report, our data shows that only 7.6% (383 sites) of the 5,010 MeCP2 binding sites are associated with CpG islands [33]. Overall, there was a significant under-representation of MYCN or MeCP2 at hypermethylated CpG islands, with 92% (984 sites) of such sites being free of MYCN and MeCP2 binding. MeCP2, as a methyl binding protein, had a higher association with hypermethylated CpG islands (49 sites; 4.6% of hypermethylated CpG islands) than MYCN (9 sites; 0.8%). Of the 313 hypermethylated sites co-occupied by MYCN and MeCP2, only 35 sites were associated with CpG islands, representing just 3.5% of the total hypermethylated CpG island loci.

**Table 1:** CpG Island Occupancy.

Dataset	No. of peaks	No. of Methylated CpG Islands	% of Peaks	% of total Methylated CpGs	P-value
MYCN MeDIP Peaks	102	9	8.82352941	0.835654596	0.0037*
MeCP2 MeDIP Peaks	312	49	15.7051282	4.549675023	0.0057*
MYCN MeCP2 MeDIP Peaks	313	35	11.1821086	3.249767874	<0.0001*
Unbound MeDIP Peaks	3323	984	29.6117966	91.36490251	<0.0001
Total MeDIP Peaks	4050	1077	26.5925926	n/a	0.00567

*Under-represented.

**Figure 3:**
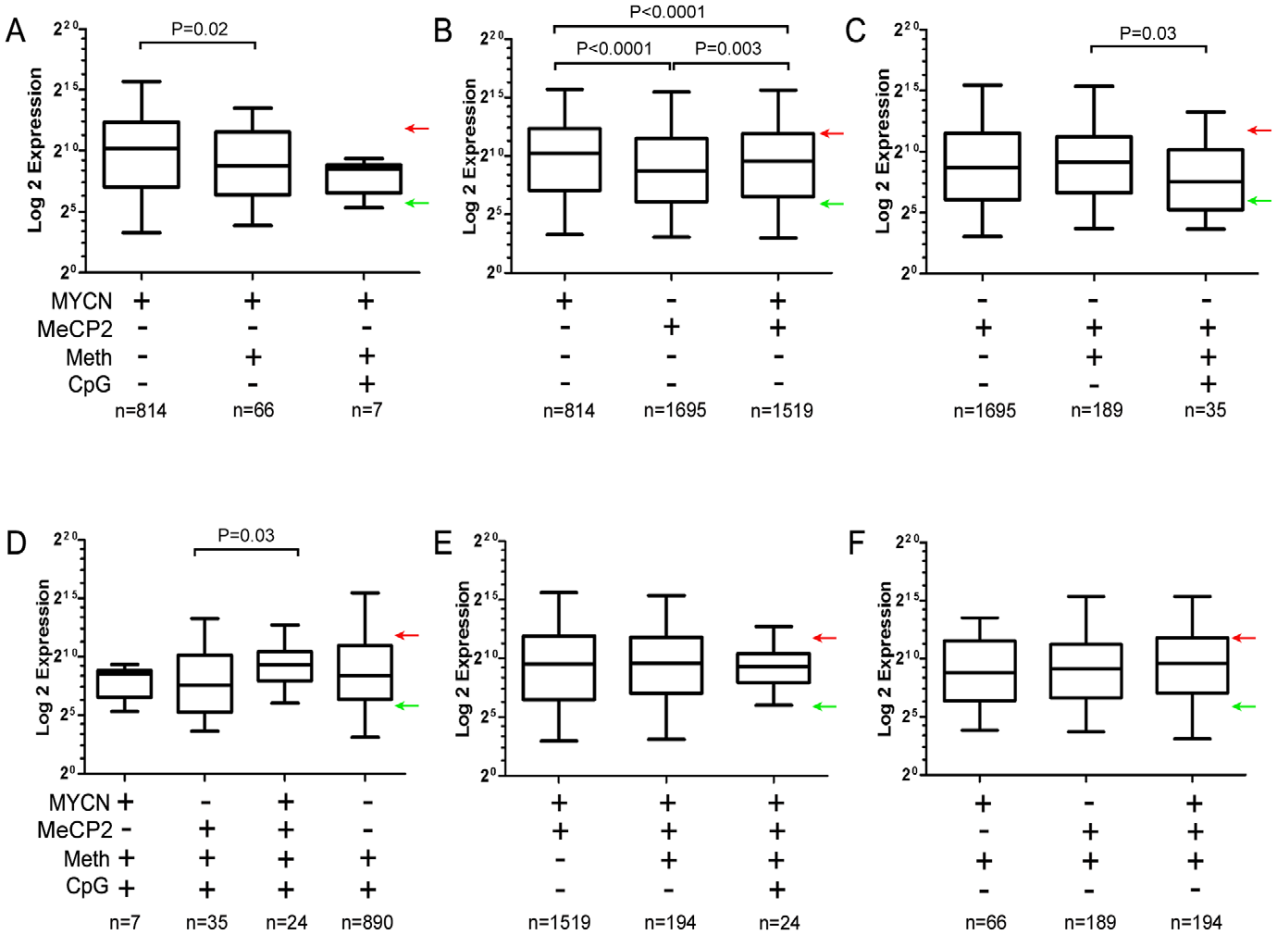
Gene expression analysis of the kelly cell line. (A) A bar chart representing the median expression levels of genes whose promoter regions are bound by MYCN. The dataset was subdivided into unique MYCN sites, hypermethylated MYCN sites and hypermethylated MYCN sites associated with CpG Islands. (B – F) Similar analysis was performed for genes having promoters with different combinations of features (MYCN +/−; MeCP2 +/−; methylation +/− and/or CpG island +/−). Red arrows and green arrows denote the upper and lower quartiles of expression from the overall gene expression microarray results. Genes with expression values which fall in the upper quartile are considered highly expressed while genes which fall in the lower quartile are considered silent or expressed at low levels. Statistical analysis was performed using Mann-Whitney nonparametric test.

Gene expression analysis of the Kelly cell line was performed using NimbleGen 4-plex 72K arrays in order to determine how MYCN and/or MeCP2 binding might influence gene expression at hypermethylated promoter sites with and without CpG islands. Results from the expression microarrays had a high correlation with selected TaqMan qPCR probes (r = 0.95; P = 0.001; Figure S4). Median expression levels of genes whose promoter regions varied in methylation status, CpG island status, MYCN or MeCP2 binding status were compared and evaluated by the Mann-Whitney nonparametric test, as illustrated in Figure 3.

Overall, hypermethylation of promoter regions had a negative impact on gene expression, as the median expression of genes at non-hypermethylated promoters bound by MYCN was 2.6 fold higher than hypermethylated promoters bound by MYCN (P = 0.02; Figure 3A). The median expression of genes with non-hypermethylated promoters that were bound by MYCN was ∼2.8-fold higher than similar promoters occupied by MeCP2 (P<0.0001; Figure 3B), consistent with MYCN having a positive impact on transcription. Overall, the median expression of genes with non-hypermethylated promoters and co-localization of MYCN and MeCP2 was intermediate between those occupied only by MYCN or MeCP2 (Figure 3B), indicating that MeCP2 interaction with MYCN can moderate gene expression. Interestingly, there was no difference in median expression for MeCP2 bound promoters that were non-hypermethylated versus those that were hypermethylated (Figure 3C); consistent with the hypothesis that MeCP2 binding in the absence of hypermethylation can have a suppressive effect. However, its repressive effects appear greater when associated with hypermethylated CpG islands within promoters relative to MeCP2 binding within promoters which do not contain CpG islands (3.0-fold; P = 0.03; Figure 3C). Among hypermethylated CpG island promoters, median gene expression was 3.4-fold higher for those co-occupied by MYCN and MeCP2 relative to those promoters only bound by MeCP2 (P = 0.03; Figure 3D), providing evidence that MYCN interaction can moderate the repressive effects of MeCP2. No other statistically significant difference in median gene expression was detected among the comparisons made (Figure 3 E and F). We conclude that MeCP2 has an overall negative impact on gene expression, while MYCN has a more positive influence. In addition, interaction of MYCN with MeCP2 appears to mitigate the negative effects of MeCP2 on gene expression, particularly at promoter regions containing CpG islands.

### Molecular Function of Downstream MYCN and MeCP2 Target Genes

**Figure 4:**
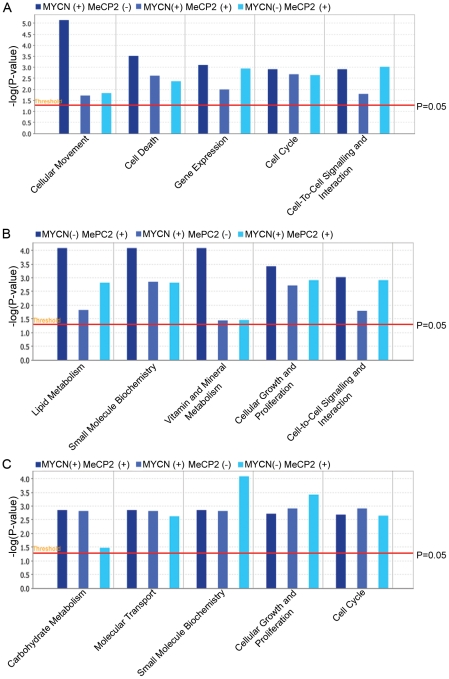
Pathway analysis of target genes. Comparison analysis of the top significant biological functions for (A) genes whose promoters are occupied by MYCN (B) genes whose promoters are occupied by MeCP2 (C) genes whose promoters are co-occupied by MYCN and MeCP2. Each panel represents the top five biological functions of each group of genes, and compares the significance of each of the functions across datasets. The significance scores for each biological function are represented as –log(P-value). The analysis was performed using the IPA software, significant biological functions were identified as having a p-value of less than P<0.05 (represented by a red bar).

In order to determine if there might be higher order functional differences between genes with promoters uniquely bound by MYCN or MeCP2 or jointly bound by both proteins, analysis of these gene subsets was carried out using Ingenuity Pathway Analysis (IPA) software. The top 5 enrichment categories for genes uniquely bound by MYCN included cellular movement, cell death, gene expression, cell cycle and cell signaling, all terms which might be expected based on numerous prior studies of MYCN (Figure 4A). Although these terms were most significantly enriched for genes uniquely bound by MYCN, they were also moderately enriched for genes uniquely bound by MeCP2 or co-bound by MYCN/MeCP2 (p<0.05). For genes whose promoters were uniquely bound by MeCP2, the top functional annotations were lipid metabolism, small molecule biochemistry, vitamin and mineral metabolism, cellular growth and cell signaling (Figure 4B). Functional terms that were enriched for genes co-bound by MYCN/MeCP2 considerably overlapped the functional terms identified in the singly bound gene sets (Figure 4C).

Although there was broad overlap between the gene pathway terms that were enriched in the three different gene sets, a more detailed analysis of the functional classes listed under the terms was informative. For example, genes involved with the cell cycle were enriched for all three categories of promoters, however, genes uniquely bound by MYCN were often involved in cell cycle progression, such as *CDC7*, *CDC23*, *CKS1B*, *NDC80* and *NUF2* whereas those uniquely bound by MeCP2 were often involved with cell cycle arrest or senescence. This observation is consistent with our finding that genes uniquely bound by MYCN are generally more over-expressed than genes uniquely bound by MeCP2. Further analysis of the broad functional category of cell death revealed that genes co-bound by MYCN/MeCP2 were enriched for the subcategories of “apoptosis of neuroblastoma cell lines” and “survival of neuroblastoma cell lines”. These subcategories included many genes known to be important in neuroblastoma pathogenesis, including *ALK, AURKA* and *BDNF* (Figure S5). As previously discussed, genes that are co-bound by MYCN/MeCP2 generally have expression levels that are intermediate between singly bound promoters. Within the cellular movement category, several genes that were identified as bound by MYCN had a functional role in cellular migration and invasion including the non-receptor tyrosine kinase PTK2. PTK2 has been implicated in NB and shown to be a direct target of MYCN [38], with higher expression levels of this protein being positively correlated with *MYCN* amplified cell lines [39]. Inhibition of this survival factor has been shown to inhibit migration and invasion of NB cell lines [40] and leads to a decrease in cell viability [41].

### Motif Analysis Reveals Differences in E-box Frequencies at Sites Bound by MYCN and MeCP2

Both c-MYC and MYCN are known to bind to the canonical CACGTG E-box motif and a number of other non-canonical motifs, although Murphy *et al.* [9] demonstrated that the frequency of MYCN binding at CACGTG sites is substantially lower than what was demonstrated for c-MYC by Zeller *et al.* [8]. In contrast to c-MYC, MYCN appears to occupy sites with a higher frequency of CATGTG and CACCTG [9]. Here, we have determined whether the frequency of E-box usage by MYCN is dependent upon its interaction with MeCP2 at regions with and without detectable levels of DNA methylation.

**Figure 5:**
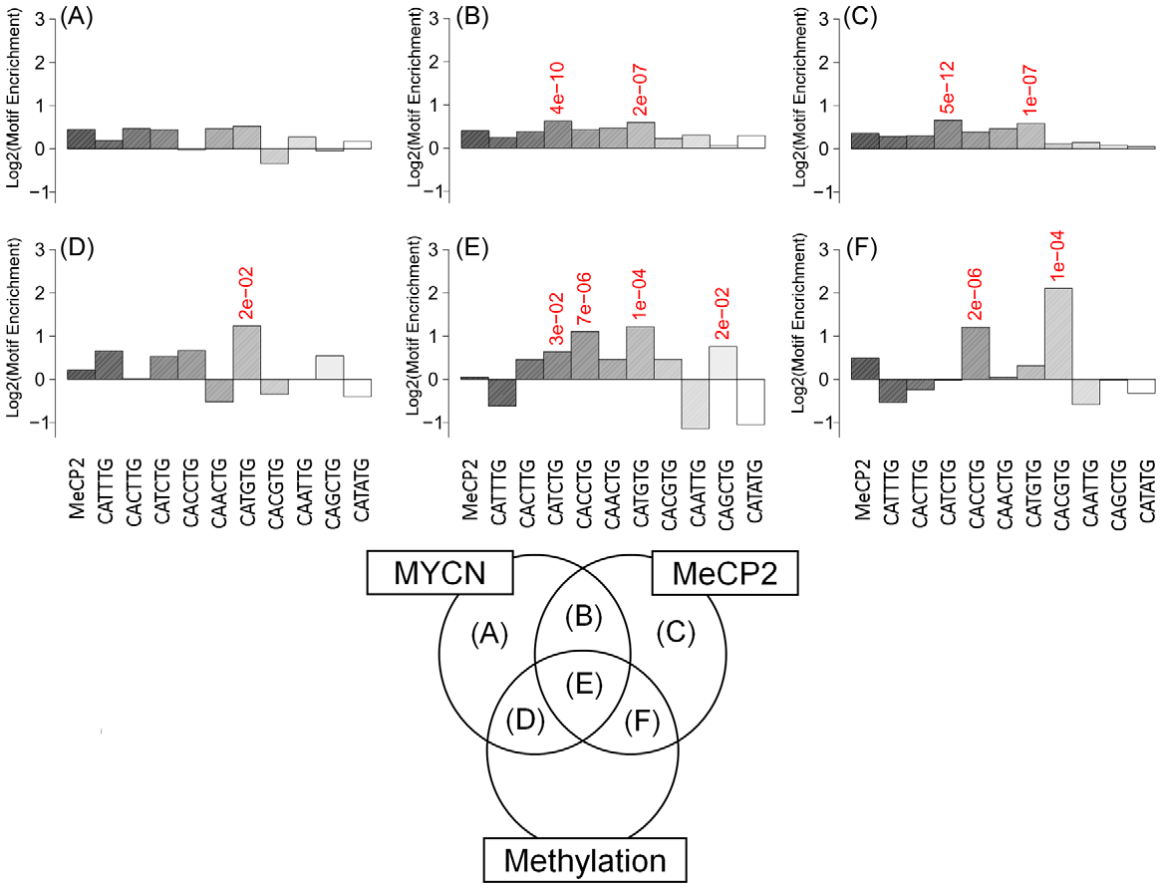
Assessment of motif enrichment at unique and commonly bound MYCN and MeCP2 sites from the promoter array data set. Here we illustrate the frequency, relative to background, of the various classes of canonical E-boxes (CANNTG) in non-methylated sites bound by MYCN alone (A), MeCP2 alone (C) and both MYCN and MeCP2 (B); and methylated sites bound by MYCN alone (D), MeCP2 alone (F) and both MYCN and MeCP2(E). We also include the putative MeCP2 binding motif proposed by Klose *et al.* (37). Motifs with 1.5 fold change over background and P<0.05 are highlighted. In non-methylated regions we see >1.5 fold-enrichment for CATCGT and CATGTG motifs. In methylated regions we see a significant preference for the CATGTG motif at locations bound by MYCN. There is a significant preference for the classical CACGTG E-box motif at methylated locations bound by MeCP2 alone.

Using supervised motif analysis of the promoter array results, we examined the frequency of all combinations of CA*NN*TG E-box motifs across the various intersections of the MeCP2, MYCN and MeDIP datasets, along with the frequency of each motif in the background data set (all sequences contained on the promoter array; Figure 5). For hypermethylated regions co-occupied by MeCP2 and MYCN, a higher frequency of CATGTG (2.3-fold above background; P<1.2e-04) and CACCTG (2.2-fold above background; P<6.5e-06) occurs, similar to our previous analysis based on MYCN binding alone [9]. There was no significant enrichment for the MeCP2 A/T rich consensus motif, as previously described by Klose et al [42]. Interestingly, the classic c-MYC binding motif CACGTG (4.3-fold above background; P<9.9e-05) was highly enriched where MeCP2 was bound to hypermethylated regions in the absence of MYCN (Figure 5F). These sites were also enriched for the CACCTG motif (2.3-fold; P<1.6e-06) and the MeCP2 A/T rich consensus motif (1.4-fold; P<9.9e-07). Similar analysis of the custom tiling array revealed that such a shift in E-box preference is not observed in hypermethylated binding sites in intergenic regions (Figure S6).

For unmethylated MYCN and MeCP2 genomic sites, there was less enrichment for E-boxes in general (1.3-fold maximum enrichment), with no clear preference for any particular E-box variant. This was also the case for regions only occupied by MeCP2 (1.2-fold).

### Bioinformatic Prediction of DNA Binding Proteins Associated with Sites Bound by MYCN and MeCP2

**Figure 6:**
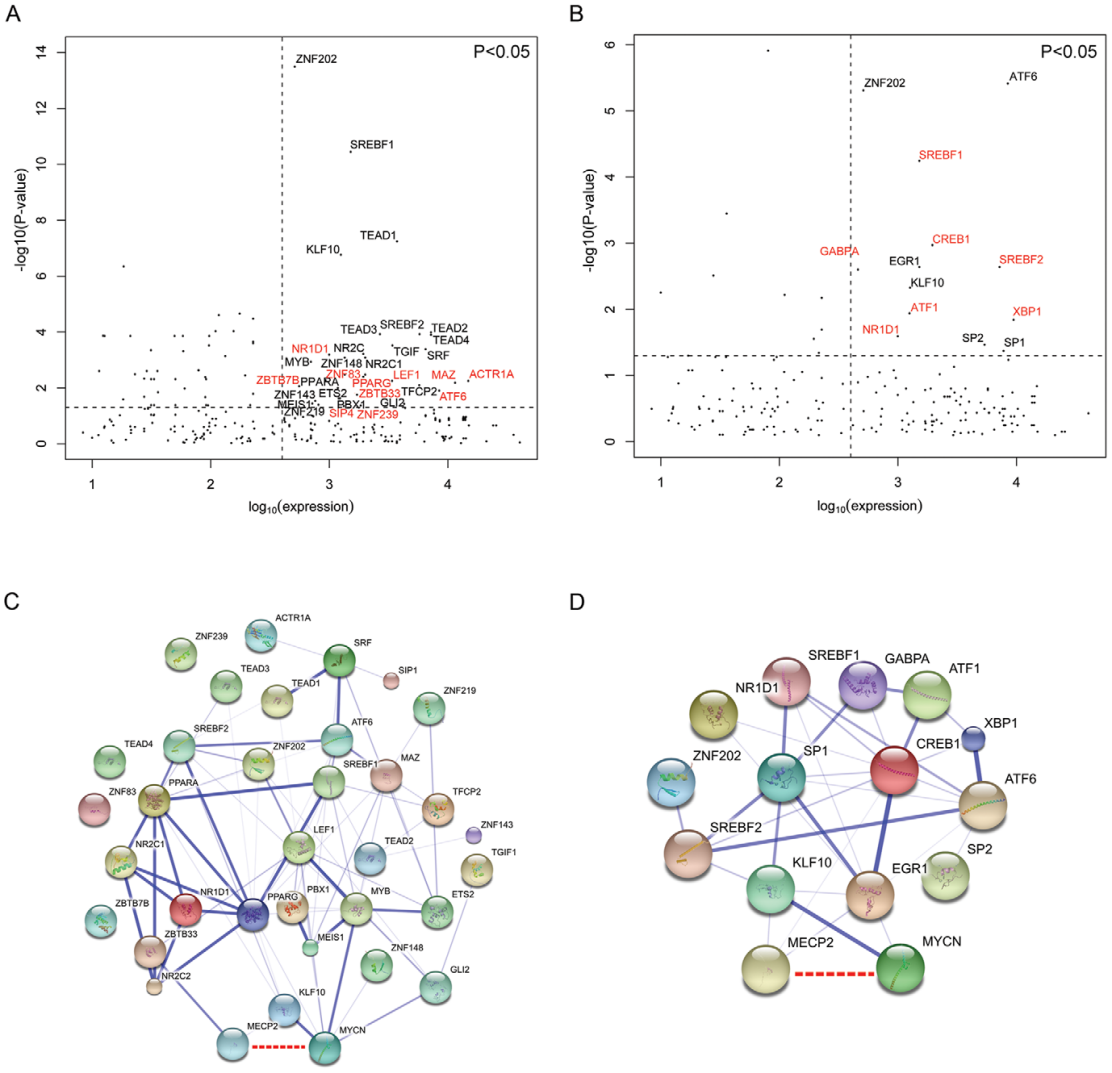
Putative association of transcription factor binding motifs at commonly bound MYCN/MeCP2 sites. For (A) and (B), plots show increasing expression of transcription factor mRNA on the X-axis and increasing enrichment for motifs on the Y-axis. For each plot, the upper right quadrant represents greater than median expression for a transcription factor and significant over-representation of the respective binding motif (P<0.05) for (A) all commonly bound MYCN/MeCP2 sites and (B) commonly bound MYCN/MeCP2 sites only at hypermethylated promoter regions. (C) and (D) illustrate potential protein interactions for the transcription factors whose motifs were over-represented (from A and B, respectively) based on available evidence from the String database. The thickness of the edges between the nodes represents the confidence of the interaction. The MYCN/MeCP2 interaction identified in this report is shown as a dashed red line.

To investigate other potential transcription factors that might be associated with MYCN/MeCP2 co-binding sites, we determined if other transcription factor binding motifs were significantly over-represented. We then cross referenced these significance values with the mRNA expression for these genes to establish which transcription factors were expressed in Kelly cells. The mRNA expression for each transcription factor was plotted against the significance of its motif enrichment [−log_10_ (P-value); Figure 6A & B]. Transcription factors with mRNA expression greater than the median and whose motifs were significantly enriched (p<0.05) in all sites co-bound by MYCN and MeCP2 are represented in the upper right quadrant of Figure 6A. A similar analysis was also carried out for MYCN/MeCP2 binding at sites that were hypermethylated (Figure 6B). By way of further external validation of this model, we examined whether any known or predicted interactions existed between these putatively co-associated transcription factors. This is illustrated in Figure 6C and D as a network in which the thickness of the lines connecting the nodes represents the confidence of the given interaction (see methods).

## Discussion

Using ChIP-chip methods we have identified a novel pattern of high frequency co-localization of the MYCN transcription factor and the MeCP2 methyl binding protein to genomic sites in neuroblastoma Kelly cells. Through a co-immunoprecipitation experiment we also demonstrate that a protein-protein interaction has occurred, but whether both, or only one protein, is binding to DNA is uncertain. The interaction of MeCP2 with another transcription factor, CREB1, has also been documented by Chahrour *et al.* [34] using mass spectroscopy analysis of protein immunoprecipitated from brain extracts of wild-type mouse with an anti-MeCP2 antibody. Consistent with the findings of Chahrour *et al.* [34], we detected a statistically significant enrichment for the CREB1 DNA binding motif at sites that were positive for MYCN, MeCP2 and methylation. Our own co-immunoprecipitation reactions revealed that MeCP2 is capable of immunoprecipitating MYCN; however MYCN was not detected in the analysis carried out by Chahrour *et al.* [34]. This disparity is likely due to the low expression levels of MYCN in the brains of adult mice, our co-immunoprecipitations were carried out on nuclear extracts from a *MYCN* amplified cell line expressing high levels of the protein.

Chahrour *et al* [34] also reported on gene expression analysis of brain tissues from *MeCP2*-null mice and from transgenic mice which over-express *MeCP2*. In contrast to the classical model, they noted that gain of MeCP2 caused significantly more activation than repression, while loss of MeCP2 caused an increase in repression and a decrease in activation. Sequential ChIP analysis of chromatin immunoprecipitated with CREB1 and MeCP2 followed by luciferase reporter and gene expression assays confirmed that the two factors associated with the promoter regions of specific genes (including *Sst* and *Mef2c*) and activated transcription. Our own analysis of gene expression indicates that many genes with MeCP2 bound promoters are transcriptionally active. However, our results also reveal the influence of MYCN, which is expressed at high levels in Kelly. As expected from a transcriptional activator, the group of genes bound by MYCN but not MeCP2 had significantly higher levels of expression (∼2.8-fold, P<0.0001) than those bound by MeCP2 alone. Groups of genes bound in combination with MYCN and MeCP2 had median expression that was intermediate to those bound by only MYCN or MeCP2. These results suggest that both proteins can act as modulators of transcription.

Previous ChIP-chip analysis of MeCP2 binding sites within the neuroblastoma cell line SH-SY5Y by Yasui *et al.* revealed that the majority of MeCP2 binding sites were unmethylated, occurred outside of CpG islands, and that downstream genes were actively expressed [33]. These results challenged the dominant model of MeCP2 as a functional repressor of transcription, acting by binding to methylated CpG dinucleotides, facilitating the recruitment of corepressors and chromatin remodeling complexes [29], [30]. Our results from the promoter arrays also indicate that MeCP2, as well as MYCN, bind to a relatively small proportion of hypermethylated promoters [11.5% of MYCN sites, 12% of MeCP2 sites]. Interestingly, similar analysis of the custom tilling array, which contained a high proportion of inter/intragenic DNA sequences, revealed a much higher association with regions of hypermethylation for both MYCN and MeCP2. [74.9% of MYCN sites, 77.6% of MeCP2 sites]. The higher frequency of MYCN/MeCP2 co-localization at hypermethylated non-promoter regions is consistent with both proteins playing a role in the maintenance of chromatin structure.

An intriguing possibility is that MYCN and MeCP2 can co-operate to regulate gene expression by altering higher-order chromatin structure. Several recent studies using chromosome conformation capture (3C) techniques have shown that distally located cis-regulatory elements can be brought into proximity to the promoter of active genes, indicating that a chromatin loop forms to allow the adjacent association of both elements. A study of the imprinted *Dlx5/Dlx6* locus using 3C combined ChIP with an anti-MeCP2 antibody, demonstrated the MeCP2 was required for the formation of a silencing chromatin loop, and that Mecp2-deficient mice had increased expression of Dlx5 and Dlx6 as a result of aberrant loop formation. The influence of MeCP2 in the formation of chromatin loops does not seem to be limited to transcriptional silencing, however, as gene expression and ChIP-chip analysis of several genes, including *JUNB,* within the NB cell line SH-SY5Y, indicate that these genes are modulated by distal and proximal MeCP2 binding sites [33]. Although the SH-SY5Y cell line is not MYCN amplified, it does over-express MYCN, suggesting that the MYCN and MeCP2 could co-operate to modulate the expression of *JUNB.* Our own ChIP-chip analysis of *JUNB* in the Kelly cell line reveals several putative overlapping MYCN and MeCP2 binding sites around the promoter region of *JUNB* which are similar to those previously described in SH-SY5Y. The mechanism that modulates chromatin looping remains poorly understood, but it is thought that changes in chromatin flexibility are necessary to allow loop formation. It has been suggested that chromatin flexibility is regulated by histone acetylation [43], as reconstituted acetylated chromatin has increased flexibility following temperature change, and is more accessible to DNase digestion [44]. MYC family members have been shown regulate histone acetylation through the recruitment of HATs such as TIP60 [45] and GCN5 [46] at a number of genetic loci. Additionally, a study by Cotterman *et al.* revealed that over 90% of the identified genomic euchromatic marks, acetylation of histone H3K9 and trimethylation of H3K4, were dependent upon MYCN expression [21].

The MYC family of transcription factors have been previously associated with specific DNA binding sequence motifs known as E-boxes. We have previously shown a preference of certain E-box motifs (CATGTG, CATTTG, CATCTG and CAACTG) in the MYCN amplified state. An E-box analysis of hypermethylated regions which were MeCP2 positive and MYCN positive, revealed enrichment for both CATGTG and CACCTG motifs. Interestingly, the classic E-box motif CACGTG was highly enriched (4.3 fold) where MeCP2 was bound to hypermethylated regions in the absence of MYCN. This is consistent with the report by Perini et al [47] that MYCN does not bind to methylated CACGTG E-boxes. In addition, the enrichment of the CACGTG motif at these MeCP2 sites would suggest a potential interaction with a basic helix-loop-helix protein such as c-MYC, a hypothesis that requires further testing. Previously, Westermann *et al.* [48] reported an anti-correlation between MYCN and c-MYC expression, although both transcription factors had a redundant core set of direct MYCN/c-MYC target genes in neuroblastoma cells.

In conclusion, our results indicate that MYCN and MeCP2 protein interact and co-localize to similar genomic sites at very high frequency. Overall, genes with MYCN and MeCP2 bound promoters have a median expression that is intermediate between genes that are uniquely bound by either MYCN or MeCP2. Our results also support the concept that MeCP2 has a repressive effect on transcriptional activity, except when it is interacting with MYCN and potentially other transcriptional activators. Currently, it is not known if this interaction contributes towards neuroblastoma disease pathogenesis or represents an important and normal process occurring in the development of the neural crest. Further studies are warranted to address these questions, particularly since the interaction of these two proteins occurs at the promoters of several genes that are important for the development of neuroblastoma, including *ALK, AURKA,* and *BDNF*.

## Materials and Methods

### Cell Culture

Kelly cells were obtained from the European Collection of Animal Cell Cultures (Porton Down, United Kingdom) and were cultured in RPMI-1640 supplemented with 2 mM glutamine, 10% fetal bovine serum and penicillin/streptomycin. The Kelly neuroblastic cell line, derived from the metastatic tissue of a female neuroblastoma patient, contains an amplification of the MYCN oncogene and expresses elevated levels of MYCN mRNA and protein [49]. A detailed DNA copy number profile of this cell line has been previously reported [50].In order to obtain sufficient material for ChIP-chip, large scale cultures were grown using hyperflask cell culture vessels (Corning Life Sciences, Corning, NY). Small scale cultures were maintained in T-75 culture flasks for qPCR, microarray expression analysis, co-immunoprecipitation and Western blot experiments.

### Chromatin Immunoprecipitation to Microarrays

ChiP-chip analysis was performed as previously described by Murphy *et al.* [9]. Briefly, protein-DNA interactions were preserved by cross-linking cells by incubation with a 1% formaldehyde solution for 10 min at room temperature. After nuclear isolation, the chromatin was sonicated to generate fragments of approximately one kilobase in length. Chromatin immunoprecipitations were performed using 10 µg of rabbit polyclonal anti-MeCP2 antibody (Abcam, Cambridge, UK) complexed with M-280 Sheep anti-Rabbit Dynabeads and a DynaMag™ magnetic particle concentrator (Invitrogen, Carlsbad, CA). Purified ChIP and input DNA was differentially labeled with Cy5 and Cy3 random primers, respectively (TriLink BioTechnologies, San Diego, CA) and co-hybridized to the HG18 two-Array Promoter set and a custom designed array manufactured by Roche NimbleGen. The promoter arrays included an average coverage of 4.7 kb around promoters, with a median probe spacing of 102 bp for all RefSeq genes, UCSC known genes and the Mammalian Gene Collection. The custom array was designed to include tiled sequence of 50-kb 5′ and 20-kb 3′ of 528 miRNAs. Scanning was performed using the Axon 4000B microarray scanner with GenePix 6.0 (Molecular Devices, Sunnyvale, CA). Image analysis and peak detection was performed using the Nimblescan Version 2.4 software and visualized using Signalmap Version 1.9. Enriched sites were identified using the normalized log_2_ ratios and the NimbleScan peak finding function. An in-built peak analysis algorithm detected significantly enriched regions that had at least 4 probes above a threshold value of log 2.0. These enriched regions were then identified as “peaks” and a false discovery rate (FDR) score was assigned. Enriched sites with a false discovery rate (FDR) of less than 0.1, which were shared across duplicate ChIP reactions were filtered using an in-house Java application and selected for further analysis. Raw log2 ratio data for array experiments are available at www.ebi.ac.uk/arrayexpress (Accession E-MTAB-621).

### Methylated DNA Immunoprecipitation to Microarrays

The protocol used was as previously described by Weber *et al.* [51] with adaptations [23]. Briefly, 5 µg of isolated DNA was sonicated to 400–800 bp in length. The sonicated DNA (4 µg) was incubated overnight with 10 µg of anti-5′ methyl-cytidine antibody (Eurogentec, Seraing, Belgium) to immunoprecipitate methylated sequences. SYBR green qPCR analysis was performed prior to microarray hybridization in order to confirm enrichment of methylated sequences (Figure S7). The methylated *H19* locus was used as the control and fold enrichment of this locus was determined relative to the unmethylated *H3B* locus [52]. Input control and MeDIP DNA samples were labeled using Cy3 and Cy5 random primers, (TriLink BioTechnologies, San Diego, CA) respectively, and co-hybridized to a custom miRNA array and to the HG18 two-Array promoter set from Roche NimbleGen. Arrays were scanned using the GenePix 4000B scanner and analyzed using the Nimblescan software Version 2.4. Normalized log_2_ ratio data was calculated and a one-sided Kolmogorov-Smirnov (KS) test using a sliding window of 750 bp was used to determine whether the probes were drawn from a significantly more positive distribution of intensity log-ratios than those on the rest of the microarray. Each probe was assigned a –log_10_ *p*-value from the windowed KS test and hypermethylated sites of enrichment were detected by searching for at least two probes with a minimum –log_10_ p-value of two. Peaks within 500 bp of each other were merged and SignalMap version 1.9 was used to visual the resulting data files.

### Gene Expression

Total RNA was extracted from Kelly cells using the RNeasy Mini Kit (Qiagen, Valencia, CA) and the complete removal of DNA was ensured via an on-column digestion of DNA using the RNase-free DNase set (Qiagen, Valencia, CA). The integrity of the RNA was confirmed using the Experion RNA StdSens Analysis Kit (Bio-Rad, Hercules, CA). Using the Superscript Double-Stranded cDNA Synthesis Kit from Invitrogen (Invitrogen**,** Carlsbad, CA), 10 µg of total RNA was used to synthesize double-stranded cDNA. The Cy3 fluorophore (Amersham Biosciences, Amersham, UK) was used to label the cDNA and 4 µg of this labeled cDNA was hybridized to the *homo sapiens* 4×72 K Gene Expression Array (Roche NimbleGen, Madison, WI). Arrays were scanned using GenePix 6.0 software on the Axon 4000B microarray scanner. Data analysis was performed using the expression robust multi-array module of Nimblescan Version 2.4. The raw data from the gene expression array experiments are available at www.ebi.ac.uk/arrayexpress (Accession E-MEXP-3121).

### SYBR Green Q-PCR Analysis

Regions of enrichment identified from duplicate ChIP-chip experiments were validated by SYBR green Q-PCR analysis. Designed PCR primers mapping to the enriched regions were manufactured by MWG (Table S2). Negative control primers mapping to un-enriched genomic regions were also designed. Q-PCR analysis was performed in duplicate on enriched DNA from anti-MeCP2 immunoprecipitations and un-enriched DNA from immunoprecipitations performed using normal rabbit IgG. The comparative Ct method was used to determine the relative level of enrichment (RQ) for each of the target regions.

### RT-PCR Validation (Taqman Probes)

Taqman gene expression assays for *CDC7* (Hs00177487_m1), *CDC25A* (Hs00947994_m1), *MeCP2* (Hs00172845_m1), *MYCN* (Hs00232074_m1), *c-MYC* (Hs00153408_m1), *TP53* (Hs99999147_a1), *TERT* (Hs99999022_m1) and *β-Actin* (HS99999903_m1) were obtained from Applied Biosystems and were used to validate the gene expression array results. Analysis was performed using the 7900HT Fast Realtime System (Applied Biosystems, Foster City, CA). The results of the gene expression array were correlated with the Ct values of gene expression probes using Spearman's rank correlation coefficient.

### Co-Immunnoprecipitation Analysis

Co-immunoprecipitations were performed using a modified version of the Universal Magnetic Co-IP Kit protocol (Active-Motif, Carlsbad CA). Briefly, 5×10^6^ Kelly cells were harvested from a T75 cell culture flask and nuclei were isolated by re-suspending the cells in a complete hypotonic buffer for 15 min on ice. After the addition of detergent, the cells were centrifuged at 14,000 x *g* in a pre-chilled microcentrifuge and the nuclear fraction was retained. The nuclei were enzymatically sheared by incubation at 37°C for 10 min after re-suspension in complete digestion buffer containing an enzymatic shearing cocktail. The reactions were stopped with the addition of 0.5 M EDTA and, after centrifugation, the supernatant containing the digested nuclei was retained. The nuclear extract (500 µg) was incubated with 5 µg of rabbit polyclonal MeCP2 antibody (Abcam, Cambridge, UK) or 5 µg of normal rabbit IgG (Santa Cruz Biotechnology, Santa Cruz, CA) on a rotating platform for 4 h at 4°C. Protein G magnetic beads were added to the reactions and the incubation was continued using the same conditions for a further hour. After incubation, the protein beads were precipitated using a magnet and after washing, the protein complexes were eluted by re-suspending the beads in 20 µl of Laemmli buffer and incubating the samples at 100° for three minutes. Each sample (10 µl) was loaded onto a 10% SDS-PAGE gel and resolved by electrophoresis.The protein was transferred to a nitrocellulose membrane which was then blocked overnight at 4°C with a 5% solution of BSA. Blots were probed with a 1 in 500 dilution of B8.4.B (Santa Cruz Biotechnology, Santa Cruz, CA) antibody, followed by a 1 in 500 dilution of Clean-Blot IP Detection Reagent (HRP) (Thermo Scientific, Waltham, MA).

### Ingenuity Pathway Analysis

Lists of genes which were identified as bound uniquely by, or in combination with, MYCN and MeCP2 using an in-house Java based software program. These lists were then imported into Ingenuity Pathway Analysis (IPA) version 8.8 (http://www.ingenuity.com). To identify the top functions of lists bound by MYCN and MeCP2, the biological functional analysis was performed on all datasets separately. The results of this analysis were compared across datasets, using Fisher's Exact test, biological functions were deemed to be significant if P<0.05.

### Transcription Factor Binding Site Analysis

DNA sequence data for the processed ChIP regions for each cell line were retrieved from the UCSC database. Phylogenetically conserved sequence between Human (HG18) and Mouse was selected for motif analysis. The occurrence of DNA binding motifs was assessed in relation to their background frequencies within the sequence tiled on the promoter array. Significance for over- or under-representation was assessed using P-values based on Chi-square test. Transcription factor motif enrichment was assessed by examining occurrence of 482 transcription factor binding motifs (from 358 transcription factors), represented by their position-weight-matrices (PWM), in the conserved DNA sequences from the various peak datasets. We then calculated over-representation of each motif compared to our promoter array background and generated a significance value from the Chi-squared test. The String database was used to retrieve information on known and predicted proteins interactions (http://string-db.org) [53]. This information is based on several types of evidence retrieved from areas such as direct experimental and text mining.

## Supporting Information

Figure S1 Pair-wise comparisons of log_2_ ratios from replicate ChIP-chip experiments from the Kelly cell line hybridised to the promoter two-array set (A & B) and the custom tiled array (C). Correlation scores above R = 0.85 were observed between replicate experiments confirming that the experiments reproducibly detected MeCP2 binding sites. (D) SYBR Green qPCR validation of positive MeCP2 binding sites. Fold enrichment of positive MeCP2 target sites is displayed. Experiments were carried out in duplicate using the standard delta delta Ct method. Results are plotted relative to a region negative to MeCP2 Binding (H3B) which is set to 1.
(TIF)

Figure S2 Identification of MeCP2 binding within the promoter regions of previously published target genes (A) *SST,* (B) *GPRIN1,* (C) *MEF2C* (D) *SGK* (E) *SNRPN* and (F) *BDNF.* The base pair position of the promoters is indicated by the scale across the top of the panels. The fluorescent intensity of the probes across replicates is expressed as log_2_ ratios and is represented as the tracks with green bars. Statistically significant MeCP2 binding sites are represented as red bars. The position of the genes and the tiled region on the array are indicated by the two lower tracks.
(TIF)

Figure S3 Pie charts representing the percentage of MeCP2 sites which are unique to the MeCP2 dataset and which overlap sites enriched for MYCN binding and regions of hypermethylation.
(TIF)

Figure S4 Gene expression validation of nimblegens 4-plex 72K arrays. Taqman gene expression probes were selected for *CDC7*, *CDC25A*, *MeCP2*, *MYCN*, *MYC*, *P53*, *TERT* and *β* *-Actin*. The Ct values for these probes were correlated with the expression values obtained for each of these genes on the array platform using Spearman's Rank Correlation Test. (Spearman's r = 0.95, p = 0.0011)
(TIF)

Figure S5 Co-localization of MYCN and MeCP2 at the promoters of neuroblastoma relevant genes. (A) promoter region of *ALK* (B) promoter region of *BDNF* (C) promoter region of *AURKA*. Data from replicate MYCN and MeCP2 ChIP-chip experiments is presented. Red bars represented regions with statistically significant over-representation of the respective proteins.
(TIF)

Figure S6 Assessment of motif enrichment at unique and commonly bound MYCN and MeCP2 sites in intergenic regions identified from the miRNA custom tiling array data set. Here we illustrate the frequency, relative to background, of the various classes of canonical E-boxes (CANNTG) in non-methylated intergenic sites bound by MYCN alone (a), MeCP2 alone (c) and both MYCN and MeCP2 (b); and methylated intergenic sites bound by MYCN alone (d), MeCP2 alone (f) and both MYCN and MeCP2(e). We also include the putative MeCP2 binding motif proposed by Klose et al (37). Motifs with 1.5-fold change over background and P<0.05 are highlighted.
(TIF)

Figure S7 qPCR showing enrichment of the imprinted *H19* gene promoter relative to the non-methylated *H3B* promoter following MeDIP from Kelly cells.
(TIF)

Table S1 Comparison of MeCP2 ChIP-chip results with Yasuai et al 2008.
(XLSX)

Table S2 PCR Primers.
(XLSX)
