# Supplementary figures and images for: Co-Localization of the Oncogenic Transcription Factor MYCN and the DNA Methyl Binding Protein MeCP2 at Genomic Sites in Neuroblastoma
Source: PLoS One. 2011 Jun 22;6(6):e21436. doi: 10.1371/journal.pone.0021436 (PMC3120883; doi:10.1371/journal.pone.0021436)

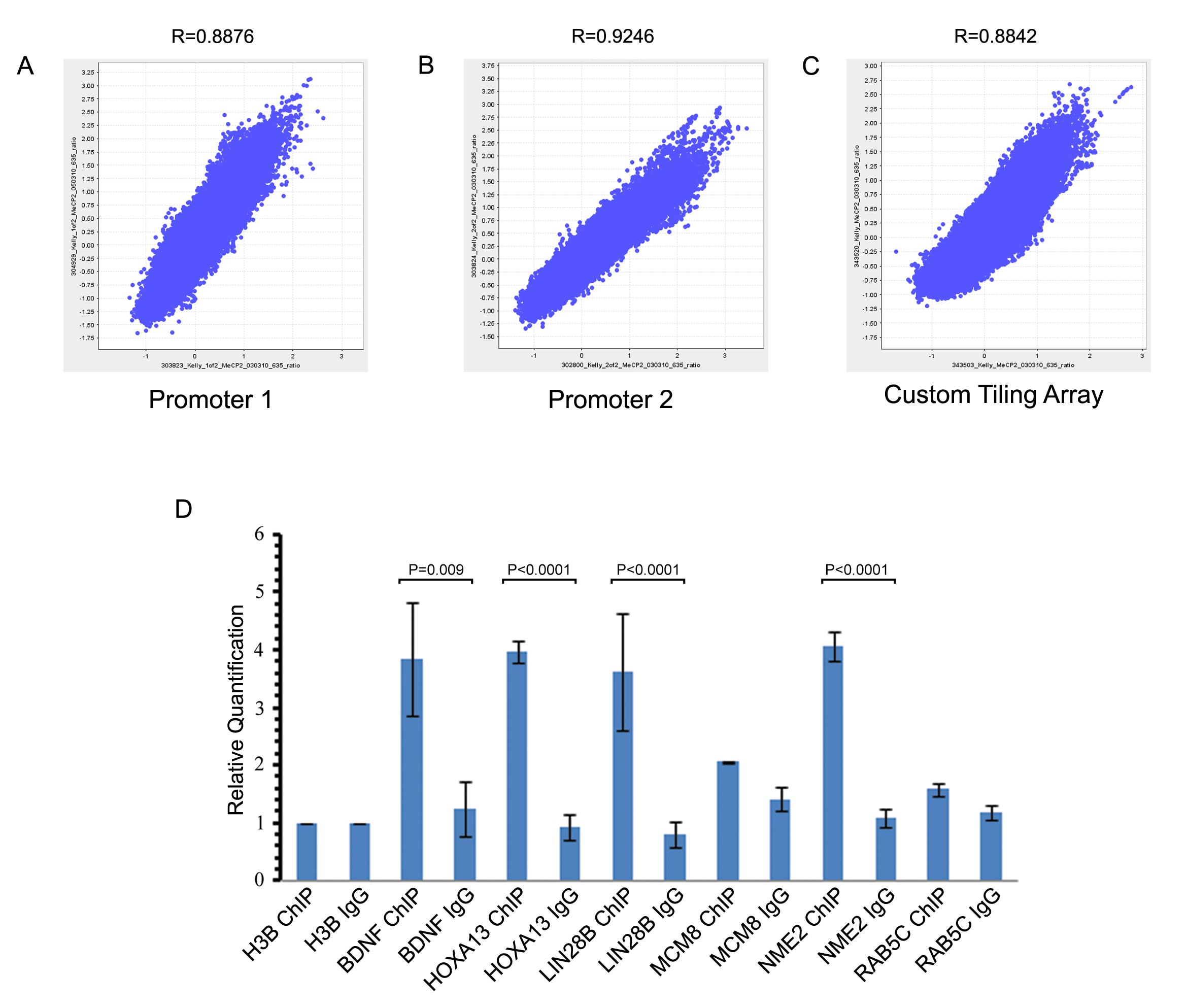

Supplement: Figure S1 — Pair-wise comparisons of log2 ratios from replicate ChIP-chip experiments from the Kelly cell line hybridised to the promoter two-array set (A & B) and the custom tiled array (C). Correlation scores above R = 0.85 were observed between replicate experiments confirming that the experiments reproducibly detected MeCP2 binding sites. (D) SYBR Green qPCR validation of positive MeCP2 binding sites. Fold enrichment of positive MeCP2 target sites is displayed. Experiments were carried out in duplicate using the standard delta delta Ct method. Results are plotted relative to a region negative to MeCP2 Binding (H3B) which is set to 1. (TIF) [file pone.0021436.s001.tif]

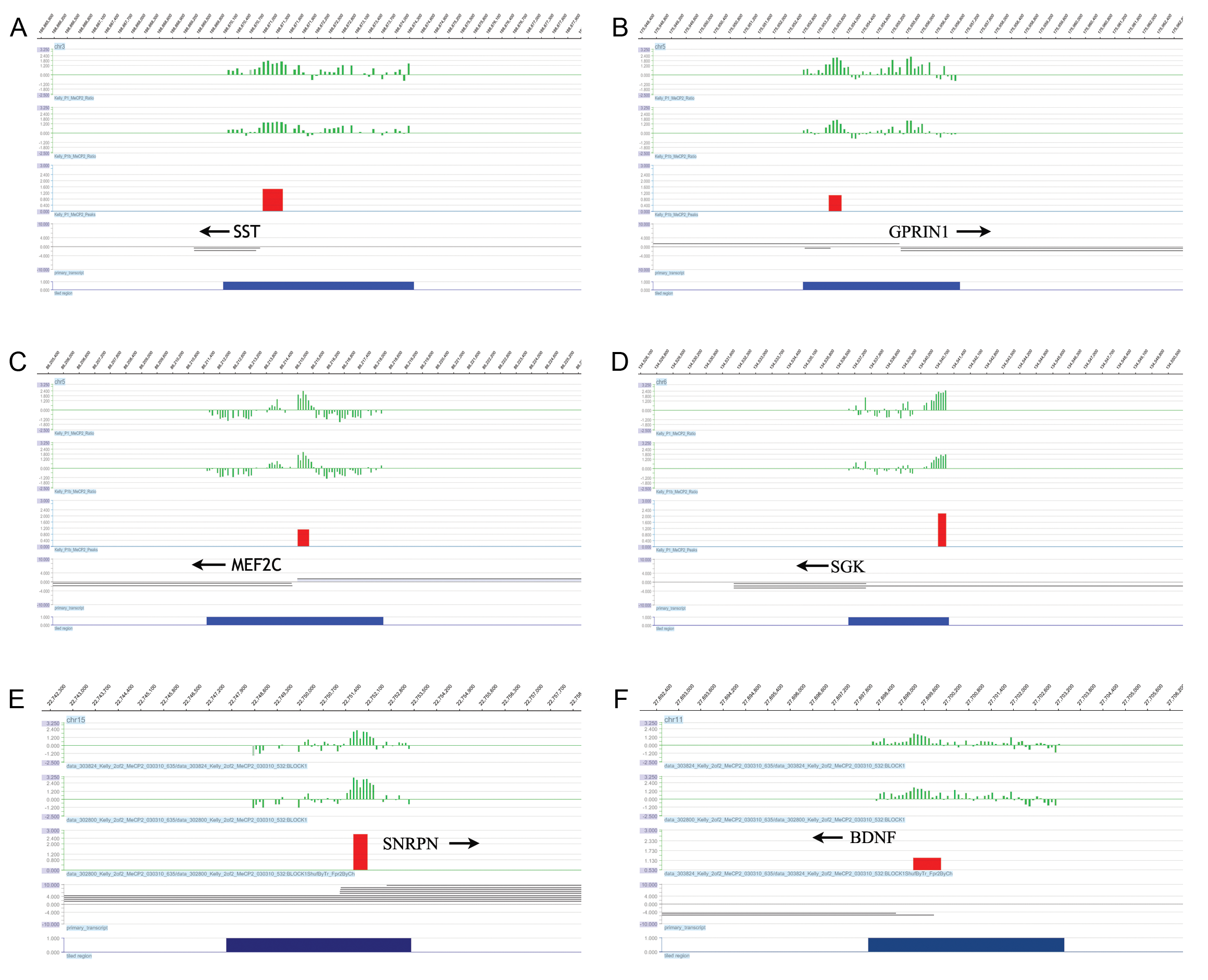

Supplement: Figure S2 — Identification of MeCP2 binding within the promoter regions of previously published target genes (A) SST, (B) GPRIN1, (C) MEF2C (D) SGK (E) SNRPN and (F) BDNF. The base pair position of the promoters is indicated by the scale across the top of the panels. The fluorescent intensity of the probes across replicates is expressed as log2 ratios and is represented as the tracks with green bars. Statistically significant MeCP2 binding sites are represented as red bars. The position of the genes and the tiled region on the array are indicated by the two lower tracks. (TIF) [file pone.0021436.s002.tif]

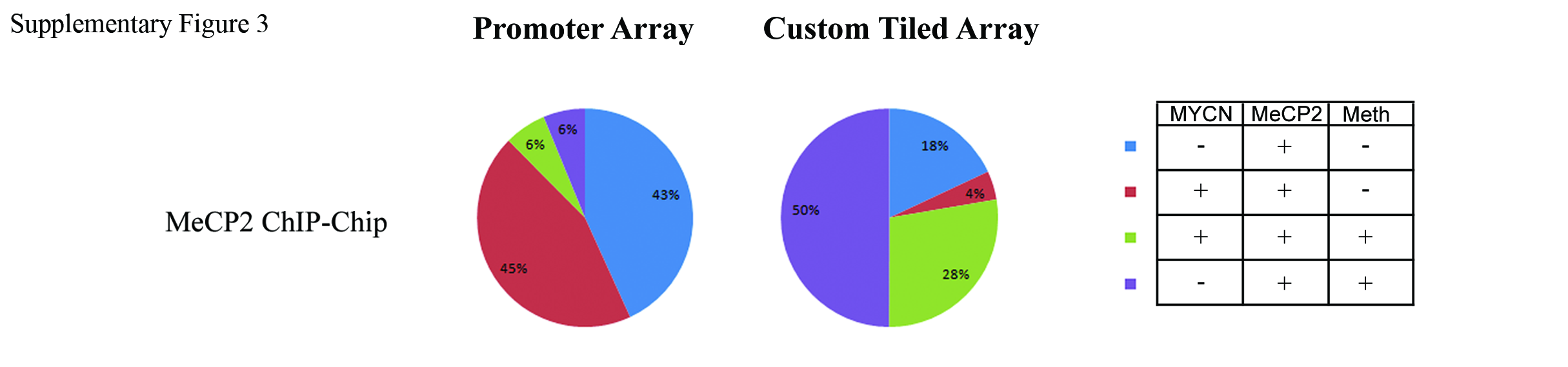

Supplement: Figure S3 — Pie charts representing the percentage of MeCP2 sites which are unique to the MeCP2 dataset and which overlap sites enriched for MYCN binding and regions of hypermethylation. (TIF) [file pone.0021436.s003.tif]

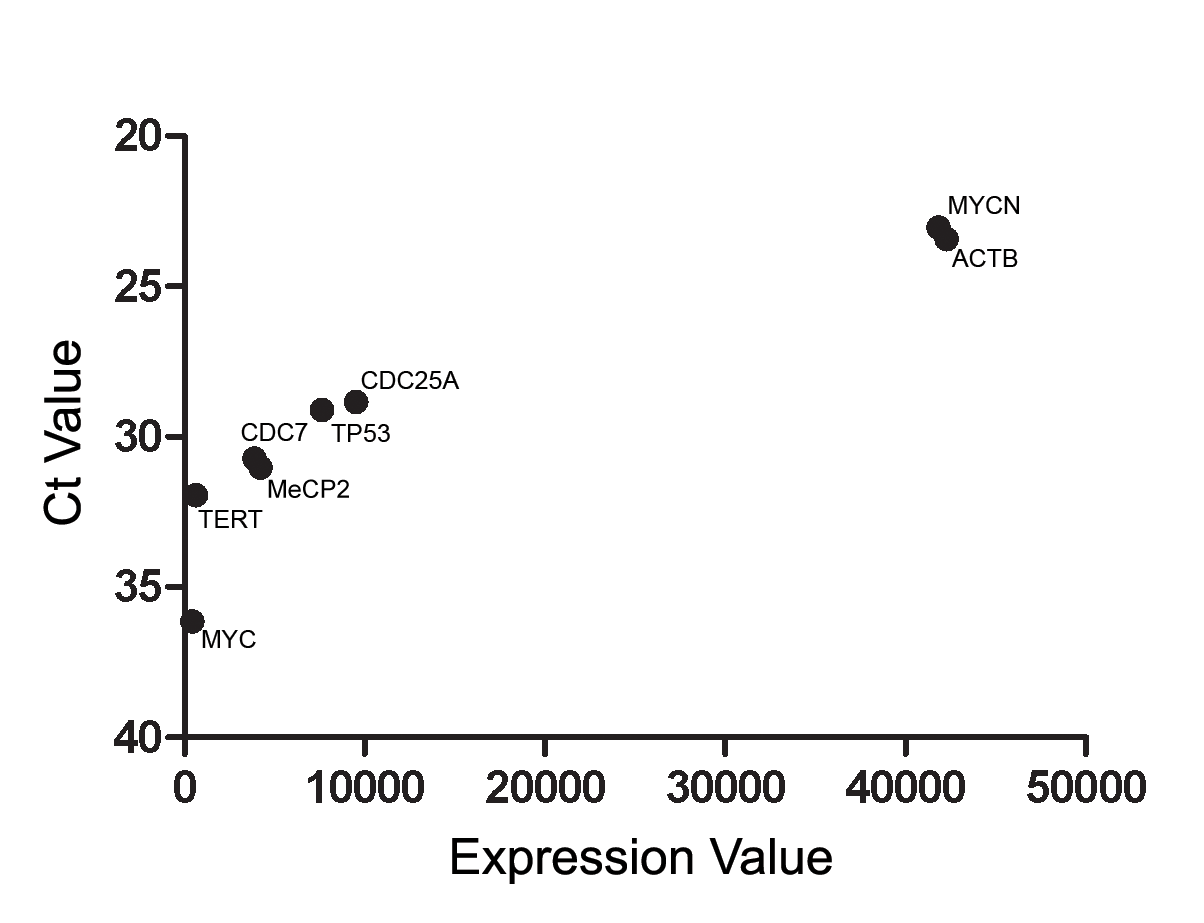

Supplement: Figure S4 — Gene expression validation of nimblegens 4-plex 72K arrays. Taqman gene expression probes were selected for CDC7, CDC25A, MeCP2, MYCN, MYC, P53, TERT and β -Actin. The Ct values for these probes were correlated with the expression values obtained for each of these genes on the array platform using Spearman's Rank Correlation Test. (Spearman's r = 0.95, p = 0.0011) (TIF) [file pone.0021436.s004.tif]

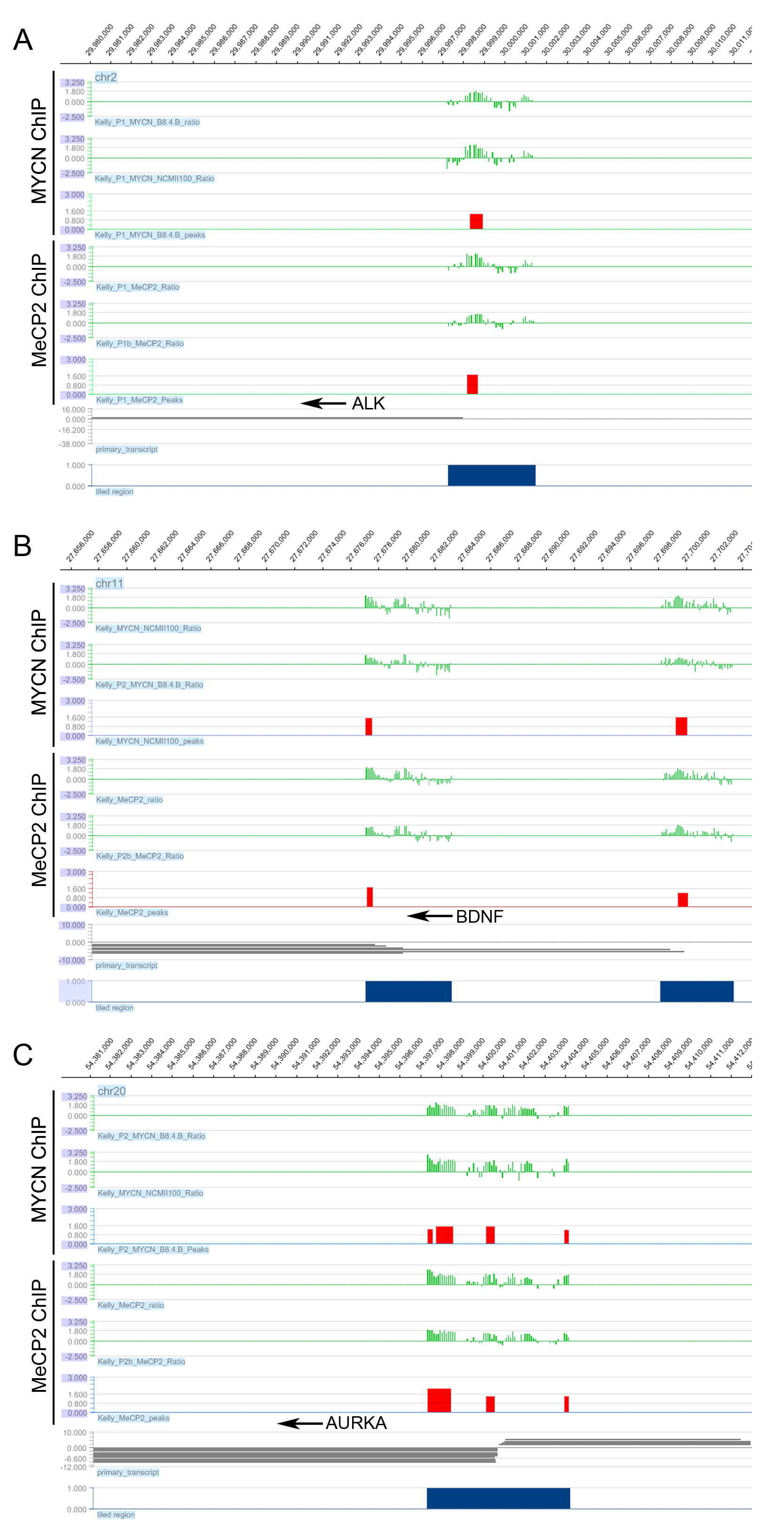

Supplement: Figure S5 — Co-localization of MYCN and MeCP2 at the promoters of neuroblastoma relevant genes. (A) promoter region of ALK (B) promoter region of BDNF (C) promoter region of AURKA. Data from replicate MYCN and MeCP2 ChIP-chip experiments is presented. Red bars represented regions with statistically significant over-representation of the respective proteins. (TIF) [file pone.0021436.s005.tif]

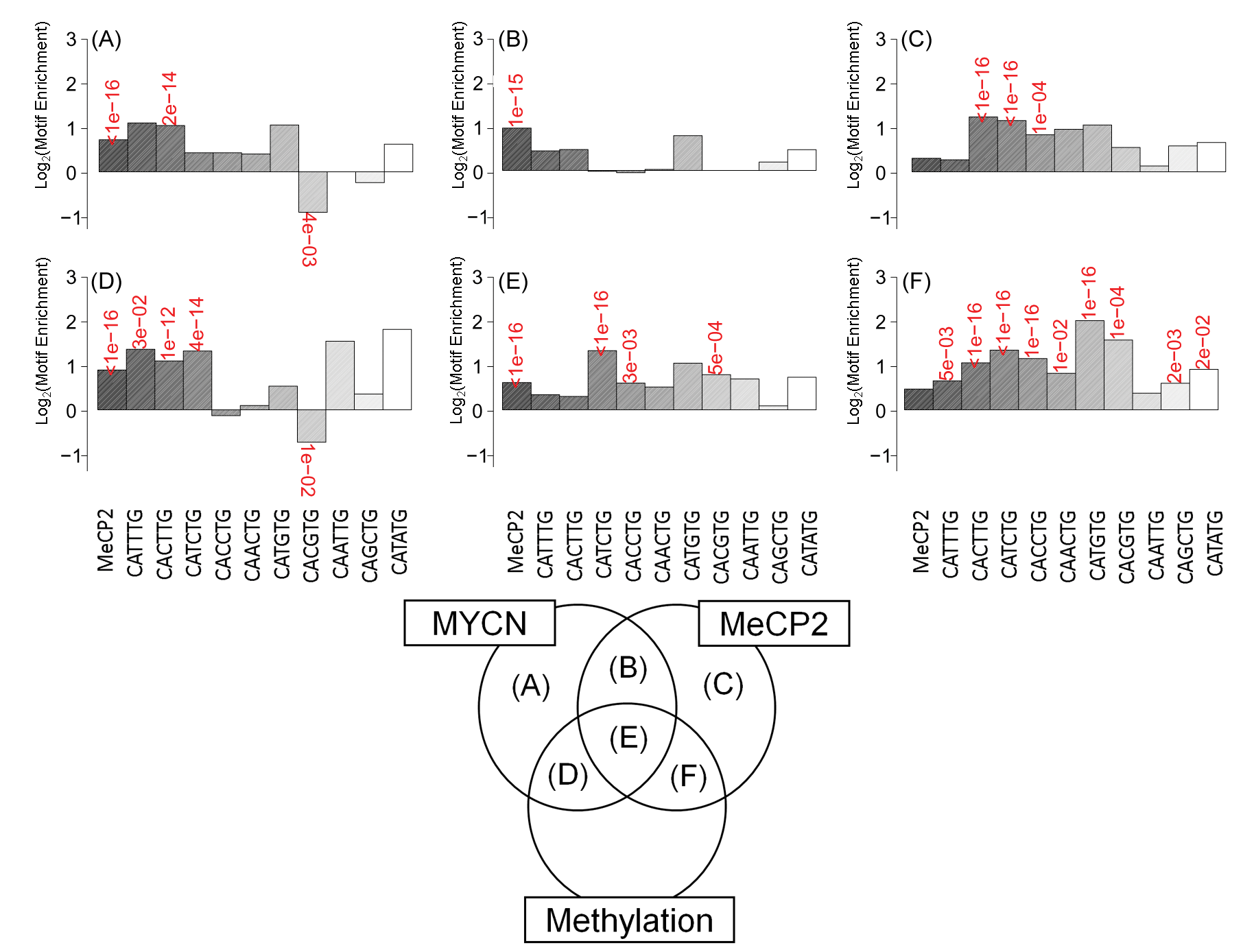

Supplement: Figure S6 — Assessment of motif enrichment at unique and commonly bound MYCN and MeCP2 sites in intergenic regions identified from the miRNA custom tiling array data set. Here we illustrate the frequency, relative to background, of the various classes of canonical E-boxes (CANNTG) in non-methylated intergenic sites bound by MYCN alone (a), MeCP2 alone (c) and both MYCN and MeCP2 (b); and methylated intergenic sites bound by MYCN alone (d), MeCP2 alone (f) and both MYCN and MeCP2(e). We also include the putative MeCP2 binding motif proposed by Klose et al (37). Motifs with 1.5-fold change over background and P<0.05 are highlighted. (TIF) [file pone.0021436.s006.tif]

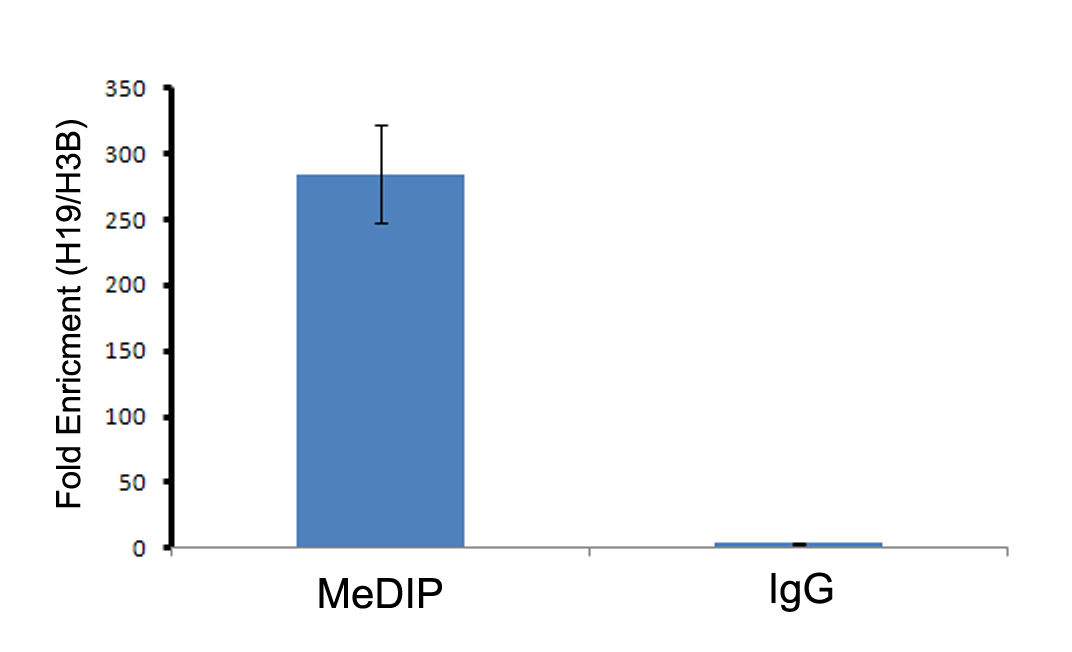

Supplement: Figure S7 — qPCR showing enrichment of the imprinted H19 gene promoter relative to the non-methylated H3B promoter following MeDIP from Kelly cells. (TIF) [file pone.0021436.s007.tif]
